# The ageing bodies study: Exploring action research methods through interdisciplinary collaboration

**DOI:** 10.1186/2193-1801-4-S2-O5

**Published:** 2015-11-27

**Authors:** Yanki Lee, Albert Siu-yin Tsang, Timothy Kin-sang Lee, Cyril Yik-ching Lee

**Affiliations:** DESIS Lab for Social Design Research, Hong Kong Design Institute and Hong Kong Institute of Vocational Education (Lee Wai Lee), Hong Kong; Engineering Discipline, Vocational Training Council, Hong Kong; Research Support Unit, Vocational Training Council, Hong Kong

## Background

The Ageing Bodies Study is a new collaborative project of the design and engineering departments of the VTC, running for 2 years from 1 July 2015. It is the first part of the Design Our Village with Elders (DOVE) project and aims to work with members of the first DesignAge HK Club, a citizen engagement unit initiated by a social design research lab at the Hong Kong Design Institute. The study's main goal is to develop a comprehensive profile of Hong Kong's ageing population, including capabilities and emotional requirements, to develop a more in-depth understanding of 'ageing in place' in Hong Kong, bring multiple perspectives to future research on ageing issues, and reveal the ingenuity of our older generations.

## Methodology

The method used in this study will be action research, a methodology that is based on the principle that people have a universal right to participate in the production of knowledge that directly affects their lives [1]. Thus, the relationship between the 'researcher' and the 'researched' is seen as inter-subjective and interactive, characterised by joint action, joint involvement and shared responsibility. As stated by Greenwood and Levin: 'A better and freer society can be built through promotion of broad participation in research processes; and support actions are expected to lead to a more satisfying situation for all the stakeholders' [2].

The experience of measuring senior citizens' physical and mental attributes will be carefully designed and refer to previous examples such as the Towards Better Design 2010 survey carried out by the i~design research team (Cambridge University's Engineering Design Centre and Psychiatry Department, Loughborough Design School at Loughborough University and the Royal College of Art's Helen Hamlyn Centre for Design) on people's ageing bodies [3]. The senior citizens involved will not be viewed as passive physical bodies but rather as research partners. The profile of Hong Kong's ageing population will be recorded and analysed and a platform will be designed to capture their valuable creativity and knowledge of everyday life so that it can be channelled towards future product development for Hong Kong society. Having fun and contributing to society will be the incentives for the participants.

## Results

Apart from collaborating with creative senior citizens in Hong Kong, the DOVE project is also researching the possibilities of interdisciplinary collaboration between experts from design and engineering disciplines, and a knowledge exchange from vocational training to industry. The database from the Ageing Bodies Study will be an important resource for the research community and the data will be applied to developing prototype innovations for Hong Kong's ageing society. The first application will be a low-cost shower for use in the confined spaces of Hong Kong senior citizens' homes.

## Conclusions

At the end of the two-year study, the DOVE project team plans to develop a profile of Hong Kong's ageing population for future research and apply the data to develop an innovative new and inclusive shower facility with the aim of promoting a safe and independent home environment for senior citizens. A participatory action methodology for this demonstration project will encourage senior citizens in Hong Kong to collaborate with younger designers and architects on future intergenerational home designs.
